# Supplementary material for: Multi‐dataset identification of innovative feature genes and molecular mechanisms in keratoconus
Source: J Cell Mol Med. 2024 Sep 19;28(18):e70079. doi: 10.1111/jcmm.70079 (PMC11412914; doi:10.1111/jcmm.70079)
Supplement: Supplementary file 2 — Material S2. [file JCMM-28-e70079-s002.zip › 7.GSEA analysis/6.SNORA74B/KEGG_PROTEIN_EXPORT.html]

Details for gene set KEGG\_PROTEIN\_EXPORT[GSEA]

|  || Dataset | SNORA74B.group.cls#h\_versus\_l |
| Phenotype | group.cls#h\_versus\_l |
| Upregulated in class | h |
| GeneSet | KEGG\_PROTEIN\_EXPORT |
| Enrichment Score (ES) | 0.2775232 |
| Normalized Enrichment Score (NES) | 0.90572214 |
| Nominal p-value | 0.6484375 |
| FDR q-value | 1.0 |
| FWER p-Value | 1.0 |
Table: GSEA Results Summary

  

Fig 1: Enrichment plot: KEGG\_PROTEIN\_EXPORT      
 Profile of the Running ES Score & Positions of GeneSet Members on the Rank Ordered List

  

| SYMBOL | TITLE | RANK IN GENE LIST | RANK METRIC SCORE | RUNNING ES | CORE ENRICHMENT || 1 | SEC61A2 | na | 4250 | 0.251 | 0.0757 | Yes |
| 2 | SRP54 | na | 11238 | 0.071 | -0.0696 | Yes |
| 3 | IMMP1L | na | 12923 | 0.048 | -0.0804 | Yes |
| 4 | SRP19 | na | 13272 | 0.044 | -0.0556 | Yes |
| 5 | SRP9 | na | 13845 | 0.038 | -0.0423 | Yes |
| 6 | IMMP2L | na | 14099 | 0.035 | -0.0222 | Yes |
| 7 | SRP68 | na | 17915 | -0.005 | -0.1282 | Yes |
| 8 | SEC61B | na | 17969 | -0.006 | -0.1253 | Yes |
| 9 | SEC11A | na | 18143 | -0.008 | -0.1243 | Yes |
| 10 | SRP14 | na | 18599 | -0.013 | -0.1273 | Yes |
| 11 | SPCS1 | na | 18933 | -0.016 | -0.1241 | Yes |
| 12 | SEC11C | na | 20181 | -0.030 | -0.1361 | Yes |
| 13 | SPCS2 | na | 20693 | -0.036 | -0.1221 | Yes |
| 14 | SRP72 | na | 21794 | -0.049 | -0.1149 | Yes |
| 15 | SRPRB | na | 22097 | -0.053 | -0.0818 | Yes |
| 16 | SEC63 | na | 22539 | -0.059 | -0.0481 | Yes |
| 17 | SEC61G | na | 22783 | -0.062 | -0.0062 | Yes |
| 18 | SPCS3 | na | 23639 | -0.075 | 0.0285 | Yes |
| 19 | SEC62 | na | 24270 | -0.085 | 0.0776 | Yes |
| 20 | SEC61A1 | na | 24365 | -0.087 | 0.1433 | Yes |
| 21 | OXA1L | na | 24890 | -0.096 | 0.2043 | Yes |
| 22 | HSPA5 | na | 25096 | -0.100 | 0.2775 | Yes |
Table: GSEA details [plain text format]

  

Fig 2: KEGG\_PROTEIN\_EXPORT      
 Blue-Pink O' Gram in the Space of the Analyzed GeneSet

  

Fig 3: KEGG\_PROTEIN\_EXPORT: Random ES distribution      
 Gene set null distribution of ES for **KEGG\_PROTEIN\_EXPORT**
